# Ultra‐small superparamagnetic iron oxide (USPIO) magnetic resonance imaging in benign mixed tumor of the parotid gland

**DOI:** 10.1002/ccr3.3477

**Published:** 2020-12-09

**Authors:** Jason M. Johnson, Abdallah S. R. Mohamed, Yao Ding, Jihong Wang, Stephen Y. Lai, Clifton D. Fuller, Rutvij Shah, Randall T. Butler, Randal S. Weber

**Affiliations:** ^1^ Department of Diagnostic Radiology The University of Texas MD Anderson Cancer Center Houston TX USA; ^2^ Department of Radiation Oncology The University of Texas MD Anderson Cancer Center Houston TX USA; ^3^ Department of Head & Neck Surgery The University of Texas MD Anderson Cancer Center Houston TX USA; ^4^ Department of Pathology The University of Texas MD Anderson Cancer Center Houston TX USA

**Keywords:** MRI, parotid, pleomorphic adenoma, ultra‐small superparamagnetic iron oxide

## Abstract

Historically USPIO has been used to help with nodal staging but not in primary tumors. The ability to concentrate USPIO may help to differentiate BMT from other types of parotid tumors.

## Graphical abstract

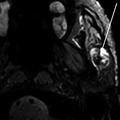

## 1 INTRODUCTION

Ultra‐small superparamagnetic iron oxide (USPIO) compounds are used to detect nodal metastases. A benign mixed tumor was evaluated with MRI including USPIO before surgery. USPIOs were significantly concentrated in the lesion. The ability to concentrate USPIO may help to differentiate benign mixed tumors from other types of parotid tumors.

Benign mixed tumors (BMT) and Warthin's tumors are the two most common tumors of the parotid glands.^1^, ^2^ Although the gold standard in lesion identification is histopathology, magnetic resonance imaging (MRI) is the preferred noninvasive method for evaluation of parotid gland tumors. On MRI, BMTs typically appear as homogeneous, T2‐hyperintense, well‐circumscribed lesions with solid contrast enhancement.^3^ Warthin's tumor, the second most common tumor of the parotid gland, typically appears as a low‐ to intermediate‐intensity lesion on T1‐weighted imaging with focal heterogeneous internal hyperintensities on T2 images. Warthin's tumors do not typically enhance.^4^, ^5^ Classically appearing BMT and Warthin's tumor can often be differentiated with a high degree of accuracy on MRI; however, the imaging appearance of these two lesions does overlap. Atypical appearances of these two parotid lesions also considerably overlap with other, less common lesions of the parotid gland, including metastatic nodal disease, mucoepidermoid carcinoma, and primary squamous cell carcinoma. In addition to MRI evaluation, computed tomography (CT), ultrasonography, and positron emission tomography (PET) each play a role in the noninvasive evaluation of parotid tumors.^5^

The information provided by MRI is routinely enhanced by the use of gadolinium chelates as a contrast agent. Newer MRI contrast agents include the ultra‐small superparamagnetic iron oxide (USPIO) compounds. Several different preparations of USPIO are approved by the US Food and Drug Administration (FDA) and are available for clinical use, including ferumoxytol, ferumoxtran‐10, and ferumoxides. These contrast preparations have different molecular weights, biologic half‐lives, and tissue distributions.^6^ Ferumoxytol is commercially available and FDA approved for the treatment of iron deficiency anemia. Thus, its use for diagnostic imaging is an off‐label use. After intravenous injection, USPIOs are taken up by macrophages or histiocytes of the reticuloendothelial cells and appear as T2 shortening on MR images because of the local magnetic susceptibility effects of these particles. Typical imaging applications of these agents include imaging of elements of the reticuloendothelial system within the gastrointestinal system, liver, spleen, and lymph nodes.^6^

Here, we describe the characteristics of USPIO imaging of a pathology‐proven BMT that was discovered incidentally during the patient's evaluation for tonsillar squamous cell carcinoma. Our case is the first, to our knowledge, to report on the MRI findings with a USPIO agent in this tumor type.

## 2 CASE DESCRIPTION

**FIGURE 1:**
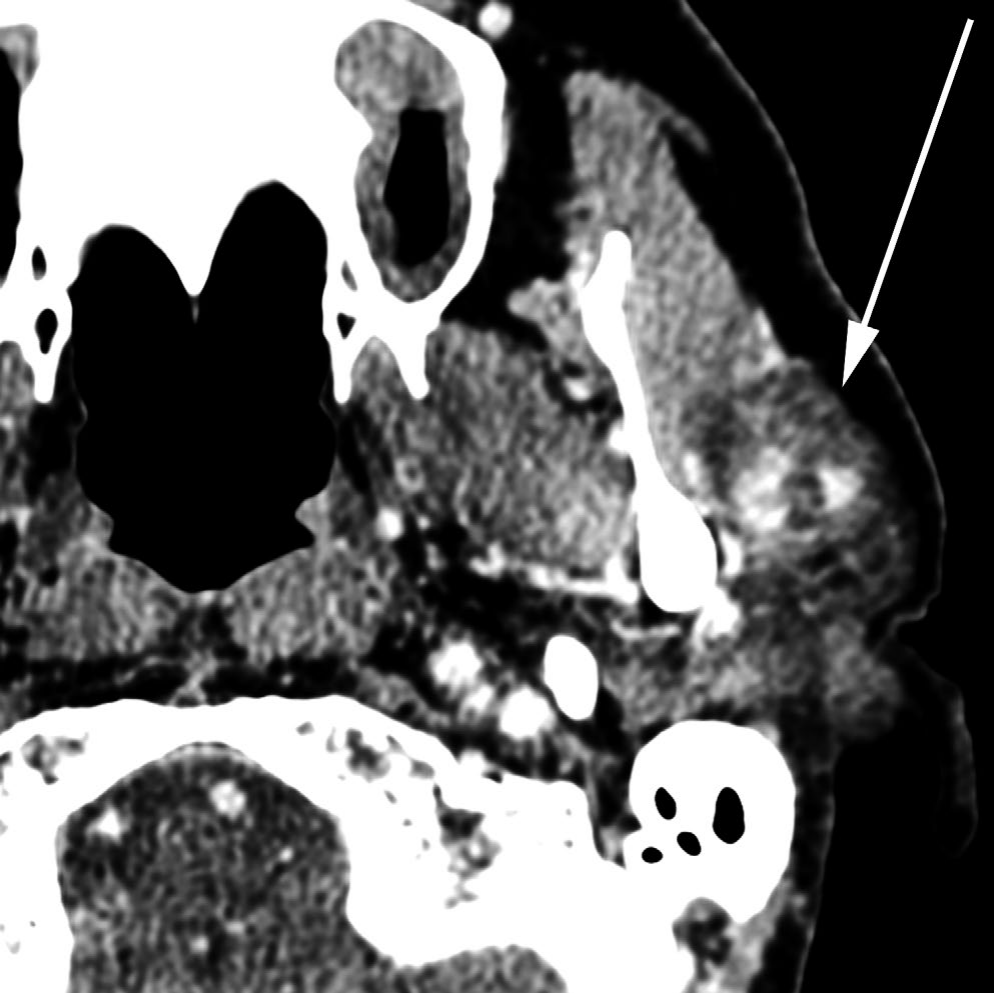
61‐y‐old man with benign mixed tumor (pleomorphic adenoma) of the left parotid gland. Initial CT of the neck with contrast reveals a heterogeneously enhancing, relatively hypointense, well‐defined lesion in the left parotid gland measuring 2.6 × 2.1 × 1.9 cm

A 61‐year‐old white male with a past medical history of chronic obstructive pulmonary disease/emphysema presented with concerns of sore throat, trismus, dysphagia, and a right tonsillar mass requiring further management. His initial CT examination revealed a 2.6 × 2.1 × 1.9‐cm left parotid mass along with the incident right tonsillar lesion and right cervical lymphadenopathy. There was no evidence of left‐sided lymphadenopathy, and the major salivary glands were otherwise normal (Figure 1).

**FIGURE 2:**
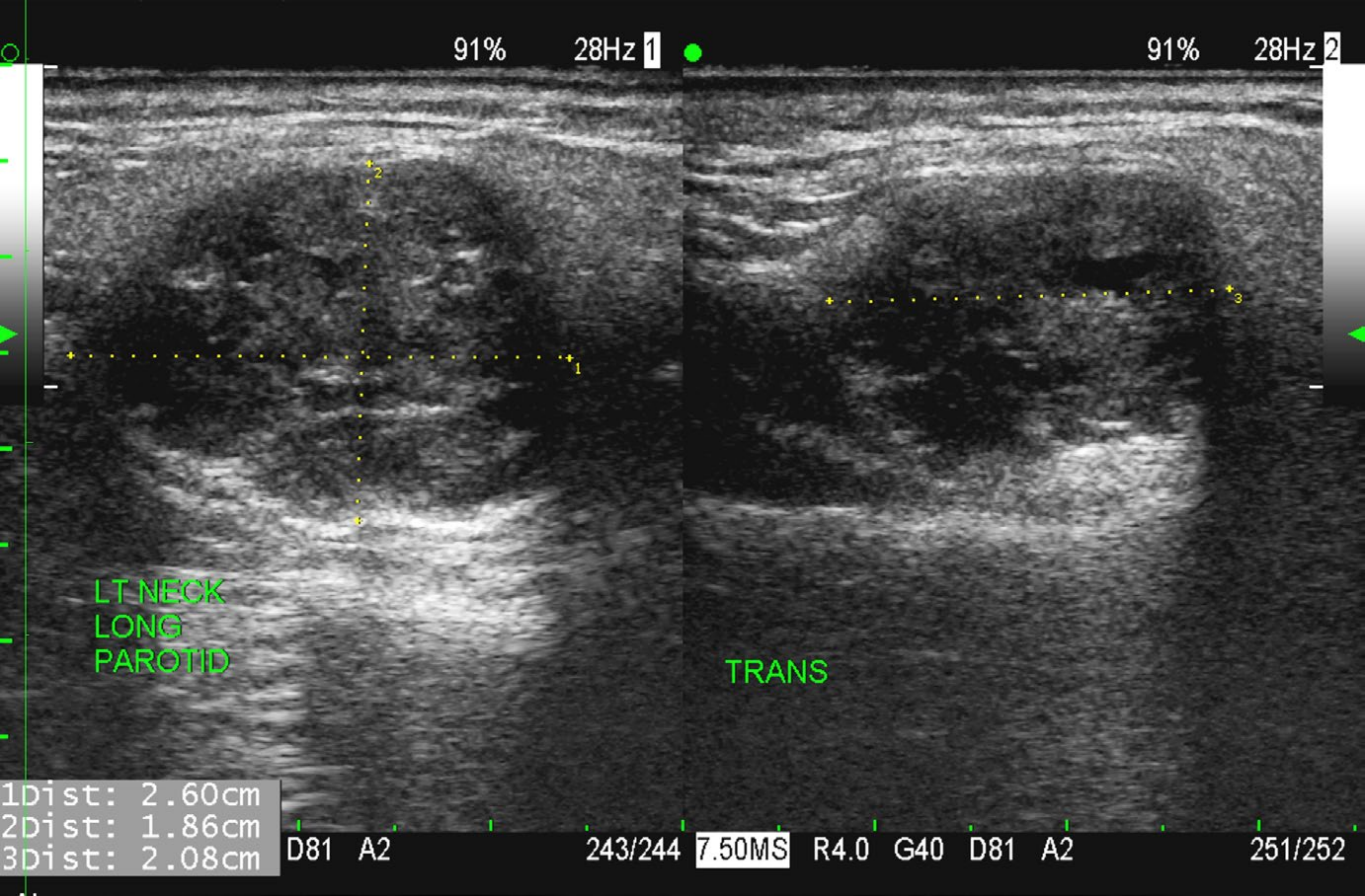
61‐y‐old man with benign mixed tumor (pleomorphic adenoma) of the left parotid gland. Ultrasound of the left parotid lesion reveals a slightly heterogeneous echotexture but a predominantly hypoechoic lesion with well‐defined borders and mild posterior acoustic enhancement. The lesion size measured with ultrasound, 2.6 × 2.1 × 1.9 cm, agreed with the size measured with CT

**FIGURE 3:**
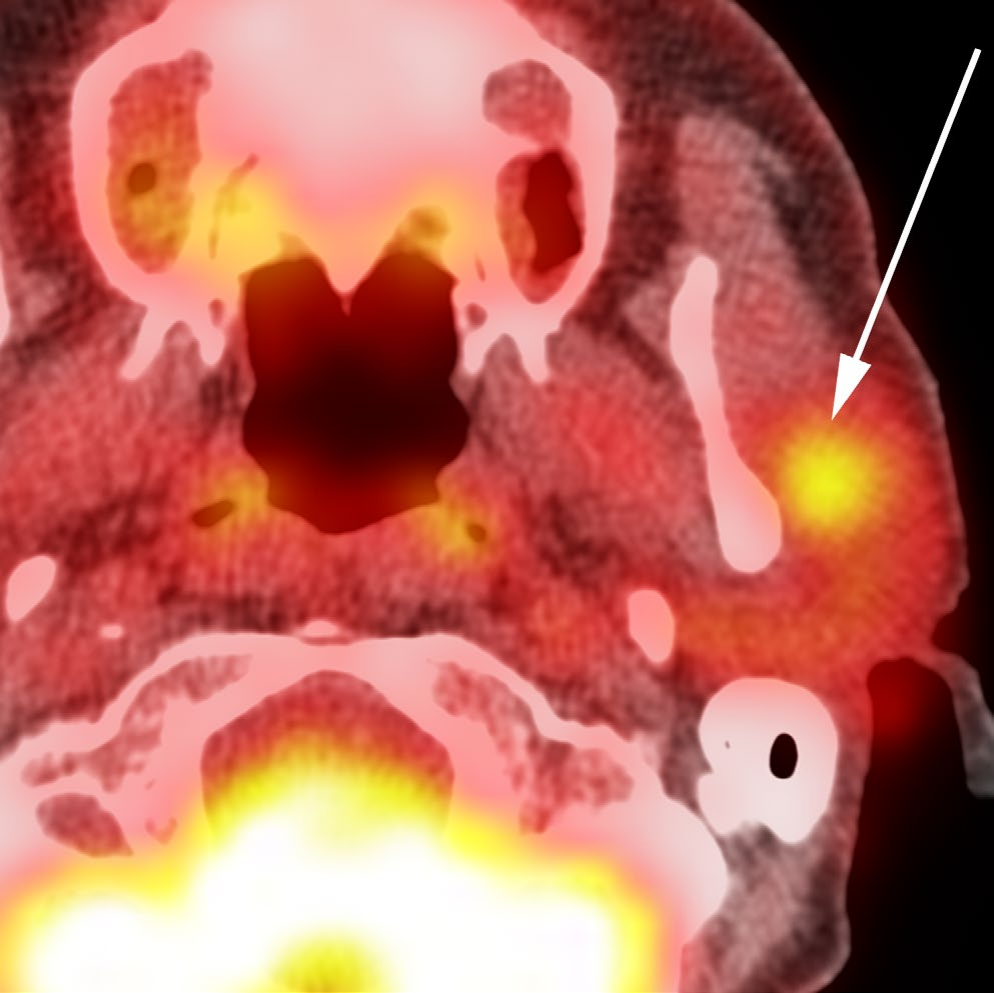
61‐y‐old man with benign mixed tumor (pleomorphic adenoma) of the left parotid gland. Fluorodeoxyglucose‐PET imaging approximately 1 h following radiotracer injection reveals modest lesion metabolism compared to background structures, with a maximum standardized uptake value of 2.9

**FIGURE 4:**
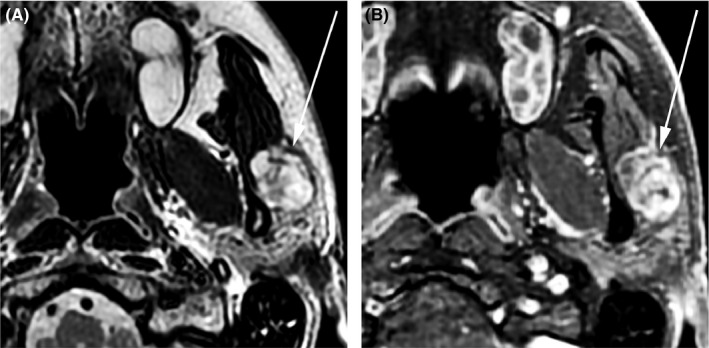
61‐y‐old man with benign mixed tumor (pleomorphic adenoma) of the left parotid gland. MRI of the neck with and without gadolinium‐based contrast reveals a well‐defined, T2‐hyperintense (A), heterogeneously enhancing (B) lesion in the left parotid gland. Minor T2‐hypointense septa are noted within the lesion

**FIGURE 5:**
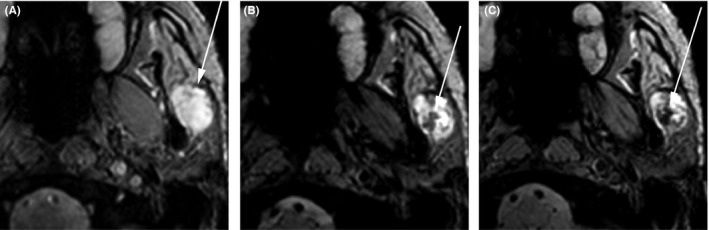
61‐y‐old man with benign mixed tumor (pleomorphic adenoma) of the parotid gland. Axial T2* gradient recalled‐echo images obtained before ferumoxytol administration (A), 30 min after ferumoxytol administration (B), and 24 h after (C) ferumoxytol administration reveal heterogeneously decreased signal within the tumor. More conspicuous areas of hypointensity are noted within the central portion of the lesion (arrows in B and C)

Results of an ultrasound study of the neck (Figure 2) and a fine needle aspiration (FNA) biopsy of the parotid mass were consistent with a diagnosis of BMT. Analysis of the FNA biopsy specimen of the nodal disease in the contralateral (right) neck confirmed squamous cell carcinoma. Staging PET/CT confirmed the local disease extension but showed no evidence of distant metastasis (Figure 3). The patient underwent a baseline 3 tesla MRI study of the neck with and without gadolinium‐based contrast, which showed a T2‐hyperintense, heterogeneously enhancing lesion in the left parotid gland measuring 25 × 17 mm (Figure 4). A follow‐up 3 tesla MRI scan of the neck performed the following day with ferumoxytol revealed the development of well‐defined areas of T2 signal hypointensity within the tumor. An 8‐mm‐diameter region of interest placed within the lesion on an axial T2* gradient recalled‐echo image revealed a decrease in mean signal intensity from 3716 (MRI signal intensity units are measureless) prior to ferumoxytol administration to 2028 (45% decrease) shortly after ferumoxytol administration and then to 1494 (60% decrease from baseline) approximately 24 hours after ferumoxytol administration. Signal intensity decreases within the left parotid gland and skeletal muscle at approximately 24 hours were 24% and 18% (Figure 5).

The patient later underwent a left total parotidectomy with excision of the deep lobe tumor and transposition of the facial nerve. Pathologic analysis of the parotid lesion revealed a typical BMT with clean margins. Multiple follow‐up CT scans of the neck, including an examination 16 months after surgery, have shown no evidence of tumor recurrence. At this writing, it has been 19 months since the patient's surgery, and he has no evidence of recurrent disease.

## 3 DISCUSSION

The parotid glands are the largest of the salivary glands and have a high density of lymph nodes within and around them in superficial and deep layers.^7^ BMT is the most common tumor of the parotid gland, accounting for 65% of all parotid gland masses.^8^ BMT usually presents as a slow‐growing asymptomatic swelling in the mandibular region. BMT is classified pathologically into myxoid (stroma‐rich), cellular (cell‐rich), and classic (balanced epithelial cells and stroma) types. The myxoid type, as seen in this case, is the most common (51% of cases), followed by the cellular (35%) and classic (14%) types; almost 80% of the total tumor volume of myxoid tumors consists of mesenchymal‐like tissue. This classification is essential for the management of these tumors, as almost two‐thirds of myxoid tumors lack a surrounding capsule, and in such cases, enucleation or local dissection can be insufficient surgical management.^9^ Both CT and MRI are used to diagnose and stage BMT.^10^, ^11^ MRI has an advantage in that it can clearly identify the lesion's margin from the surrounding parenchyma and is more useful for staging the rare but significant finding of the perineural spread of disease.

Ultra‐small superparamagnetic iron oxide agents are taken up by macrophages and histiocytes in the reticuloendothelial system, which is concentrated in organs such as the liver, spleen, and lymph nodes. The relatively high local concentration of these iron particles creates T2 and T2* shortening.^6^ Ultra‐small superparamagnetic iron oxide compounds are also helpful in differentiating normal and reactive lymph nodes from metastatic nodes. Normal and reactive nodes accumulate USPIO compounds because of their normal reticuloendothelial systems and therefore show characteristic T2 changes. In contrast, metastatic nodes lose their ability to accumulate these compounds because their normal reticuloendothelial system is replaced by the cancerous cells.^6^

In our case, the detection of heterogeneous T2 and T2* hypointensity in a BMT after the administration of USPIO was an unexpected finding, but it demonstrated that this ability to concentrate USPIO could potentially differentiate BMT from other types of parotid tumors. If this concept were supported in future studies, it would make the diagnostic use of USPIO a useful adjunct imaging technique for salivary gland tumors.^6^

A potential explanation for this finding of heterogeneous iron particle uptake is that BMTs are considered the “heroes of a thousand faces” in pathology. No two BMTs look exactly alike under the microscope; for that reason, BMT was traditionally called pleomorphic adenoma, although in this context the term *pleomorphic* refers to the variability of cells within the tumor (a feature often seen in malignancies), not the variability of entire tumors. BMTs consist of both ductal and myoepithelial cells and run a gamut from very cellular (with both cell types or predominantly myoepithelial cells) to very cell‐poor and mostly composed of stroma, and the same tumor can have different areas at different spots on this spectrum. We suspect that this variability may explain the heterogeneous uptake of USPIO.

Another possible explanation, in our case, is that the particles are taken up by macrophages, which were present as part of chronic inflammation following tissue injury caused by the FNA biopsy conducted before the baseline MRI. However, the biopsy was done 40 days ahead of the USPIO MRI scan, and chronic inflammatory cells are typically cleared from an FNA site within that time span.

## 4 CONCLUSION

Our case serves as a guide for future clinical research to study the role of USPIO agents in imaging parotid and other salivary gland tumors. Further work is needed to confirm these findings in a larger series including varied types of salivary gland tumors.

## CONFLICT OF INTEREST

None declared.

## AUTHOR CONTRIBUTIONS

JMJ: conceptualized the study, curated the data, wrote the original draft, and reviewed and edited the final manuscript. ASRH, YD, JW, RS, RTB: participated in data curation, methodology, and edited the final manuscript. SYL and CDF: participated in conceptualization, provided project administration, resources, supervision, and reviewed and edited the final manuscript. RSW: provided investigation, resources, supervision, validation, and reviewed and edited the final manuscript.

## ETHICAL APPROVAL

This study has been conducted under guidelines established by the local Institutional Review Board that governs the activity of case reports.

## DISCLOSURE

Authors do not have institutional and financial conflicts including employment, consultancies, stock ownership, honoraria, paid expert testimony, patents or patent applications, and travel grants. Dr Fuller has received funding from the National Institute for Dental and Craniofacial Research Award (1R01DE025248‐01/R56DE025248) and Academic‐Industrial Partnership Award (R01 DE028290), the National Science Foundation (NSF), Division of Mathematical Sciences, Joint NIH/NSF Initiative on Quantitative Approaches to Biomedical Big Data (QuBBD) Grant (NSF 1557679), the NIH Big Data to Knowledge (BD2K) Program of the National Cancer Institute (NCI) Early Stage Development of Technologies in Biomedical Computing, Informatics, and Big Data Science Award (1R01CA214825), the NCI Early Phase Clinical Trials in Imaging and Image‐Guided Interventions Program (1R01CA218148), the NIH/NCI Cancer Center Support Grant (CCSG) Pilot Research Program Award from the UT MD Anderson CCSG Radiation Oncology and Cancer Imaging Program (P30CA016672), the NIH/NCI Head and Neck Specialized Programs of Research Excellence (SPORE) Developmental Research Program Award (P50 CA097007) and the National Institute of Biomedical Imaging and Bioengineering (NIBIB) Research Education Program (R25EB025787). Dr Fuller has also received direct industry grant support, speaking honoraria, and travel funding from Elekta AB.

## Data Availability

The authors confirm that the data supporting the findings of this study are available within the article.
